# Effects of Oxygen–Ozone Therapy and Physiotherapy on Functioning in Patients with Chronic Non-Specific Neck Pain: A Prospective Double-Arm Pilot Study

**DOI:** 10.3390/jfmk11020227

**Published:** 2026-06-03

**Authors:** Alessandro de Sire, Andrea Parente, Andrea Demeco, Emanuele Prestifilippo, Martina Cocco, Stefano Fasano, Klemen Grabljevec, Umile Giuseppe Longo, Nicola Marotta, Antonio Ammendolia

**Affiliations:** 1 Physical and Rehabilitative Medicine, Department of Medical and Surgical Sciences, University of Catanzaro “Magna Graecia”, 88100 Catanzaro, Italy; alessandro.desire@unicz.it (A.d.S.); ammendolia@unicz.it (A.A.); 2 Research Center on Musculoskeletal Health, MusculoSkeletalHealth@UMG, University of Catanzaro “Magna Graecia”, 88100 Catanzaro, Italy; 3 Physical and Rehabilitative Medicine, ASL Cagliari, 09047 Cagliari, Italy; 4 Department for Rehabilitation After Central Nerve System Lesions, University Rehabilitation Institute, 1000 Ljubljana, Slovenia; 5 Fondazione Policlinico Universitario Campus Bio-Medico, Via Alvaro del Portillo 200, 00128 Roma, Italy; 6 Research Unit of Orthopaedic and Trauma Surgery, Department of Medicine and Surgery, Università Campus Bio-Medico di Roma, Via Alvaro del Portillo 21, 00128 Roma, Italy; 7 Physical and Rehabilitative Medicine Unit, Department of Experimental and Clinical Medicine, University of Catanzaro “Magna Graecia”, 88100 Catanzaro, Italy

**Keywords:** oxygen-ozone injections, neck pain, Mckenzie physical therapy exercises, back school

## Abstract

**Objectives**: This study aimed to evaluate the effects of a combined treatment consisting of O_2_O_3_ injections and McKenzie-based physiotherapy exercises, compared to a Control group treated with O_2_O_3_ injections and a Back School physiotherapy program, in reducing pain and disability in individuals with chronic non-specific neck pain. **Methods**: In this prospective double-arm pilot study, patients with chronic non-specific neck pain and a Numerical Rating Scale (NRS) > 4 were enrolled. All patients received eight weekly sessions of O_2_O_3_ injections (10 μg/mL, 10 mL total, and 2 mL bilaterally into the cervical paravertebral muscles). Patients were then randomly assigned (1:1 ratio) to either an experimental group receiving McKenzie physiotherapy or a Control group undergoing Back School techniques, with five sessions per week over two weeks. Outcome measures included the Neck Disability Index (NDI), NRS, EuroQol-5D-3L (EQ5D3L), and EuroQol Visual Analog Scale (EQ-VAS). **Results**: A total of 41 patients were included and divided into two groups: Back School (n = 21; mean age: 63.9 ± 13.4 years) and McKenzie (n = 20; mean age: of 57.3 ± 12.9 years). Both groups showed significant improvement in NDI, NRS, EQ5D3L, and EQ-VAS following the O_2_O_3_ injection cycle (∆T0–T1 *p* < 0.001). The subsequent addition of physical therapy led to further improvements across all outcomes in both groups (∆T1–T2 *p* < 0.001), with the McKenzie group showing slightly greater benefits, despite the lack of significant differences. **Conclusions**: This study demonstrated the effects of combining O_2_O_3_ injections with either McKenzie or Back School therapy in improving pain, disability, and quality of life in patients with chronic non-specific neck pain.

## 1. Introduction

Neck pain is defined as pain localized between the superior nuchal line and the spinous process of the first thoracic vertebra [1]. Chronic non-specific neck pain represents a major public health issue, with significant implications for healthcare costs, productivity loss, and decreased quality of life. It is considered the fourth leading cause of disability worldwide, with a high prevalence in both the general and working populations [2].

The latest epidemiological analysis indicates that neck pain has an annual incidence affecting 30% of the general population [3]. It is estimated that approximately 75% of people have had at least one episode of neck pain during their lifetime. The prevalence increases with skeletal maturity, peaking in middle age, and women are more affected than men [4,5].

The etiology includes cervical vertebral fractures, intervertebral disk herniations, spinal cord and nerve injuries, and inflammatory or infectious diseases, often correlated with age-related musculoskeletal diseases [6,7]. In most cases, acute musculoskeletal neck pain is self-limiting. However, the 50% of patients continue to feel recurrent episodes of pain, demanding appropriate treatment [8]. Therefore, it is crucial to provide patients with proper management for pain and cervical functioning, and thus a better quality of life.

Various conservative approaches have been developed for neck pain, including the use of steroidal and non-steroidal anti-inflammatory drugs (NSAIDs), physical therapies and physical agent modalities, mesotherapy, and acupuncture [9]. Although there is considerable variability in the therapies and across current guideline recommendations, several systematic reviews have highlighted how non-pharmacological interventions may be safer and effective in reducing pain and disability compared to pharmacological treatment [9,10].

Therefore, in recent years, there has been a growing interest in oxygen–ozone therapy (O_2_O_3_) injections to manage various musculoskeletal disorders, as part of a rehabilitative program to reduce pain and improve function [11,12,13,14,15,16,17,18,19,20]. Intramuscular paravertebral injections of O_2_O_3_ might provide mechanical and anti-inflammatory effects through the modulation of cytokines and superoxide dismutase levels, improving perineural microcirculation [11,12,13,14,15,16,17,18,19,20]. Particularly in neuropathic pain, the antiedematous and rheological properties of O_2_O_3_ may enhance perineural microcirculation; this improvement might exert a trophic effect on the nerve root, effectively counteracting hypoxia, resulting from both arterial compression and localized venous congestion [11,12,13,14,15,16,17,18,19,20].

In this scenario, intramuscular paravertebral injections of O_2_O_3_ might play a role also in neck pain, showing significant pain relief, function enhancement, and quality of life improvement, considering potential beneficial effects in treating myofascial pain and trigger points, thus promoting pain relief through the stimulation of antinociceptive pathways [11,12,13,14,15,16,17,18,19,20].

Nevertheless, a multidisciplinary approach is recommended for back pain, including physical therapy, pharmacological treatment, education, and exercise [21,22,23,24]. In particular, exercises might play a key role, mostly when neck pain is associated with cervical muscular weakness, principally affecting the deep neck flexors, and with postural misalignment, often characterized by a forward head position; these factors can contribute to compressive loading of the cervical spine [21,22,23,24]. In these cases, it is crucial to provide patients with strengthening of the cervical spine muscles and education on the improvement of the postural alignment [21,22,23,24]. Active exercises typically involve flexion–extension movements of the head, lateral inclinations, and rotations, followed by stretching exercises, particularly targeting the scalene muscles [21,22,23,24]. In this context, McKenzie reported interesting results improving pain and disability related to neck pain [25,26,27,28], with exercises relying on patients engaging in self-directed repeated movements and sustained positions, reducing pain and disability and improving spine mobility (with a different rationale from manipulations).

Another educational and exercise-based approach for neck pain is the Back School method, which combines postural education, ergonomics, and specific exercises to improve spinal function and prevent recurrence of pain. The rationale behind this method lies in increasing patients’ awareness and active participation in managing their spinal health, promoting functional autonomy and long-term benefits [29,30].

However, to date, there have been no studies investigating the combination of O_2_O_3_ and different physiotherapy approaches to treat patients with neck pain.

Therefore, this study aimed to evaluate the effects of a combined treatment consisting of O_2_O_3_ injections and McKenzie or Back School physiotherapy for pain relief and disability reduction in patients with chronic non-specific neck pain.

## 2. Materials and Methods

### 2.1. Participants

In this prospective double-arm pilot study, patients affected by chronic non-specific neck pain were enrolled at the Physical Medicine and Rehabilitation Unit of the “Renato Dulbecco” University Hospital of Catanzaro, Italy, from March 2024 to November 2024. Inclusion criteria were: (a) diagnosis of chronic non-specific neck pain lasting at least 12 weeks, associated with myofascial pain; (b) Numeric Rating Scale (NRS) score ≥ 4; (c) presence of active trigger points in the upper trapezius muscles; (d) age over 18 years; (e) Body Mass Index (BMI) < 30 kg/m^2^; (f) patients consenting to interrupt any current treatment with NSAIDs, opioids, corticosteroids, muscle relaxants, or any other therapy that could interfere with the study evaluations; and (g) patients who have not taken antidepressants and/or benzodiazepines for at least 60 days. In addition, (h) women of childbearing potential must have a negative pregnancy test and agree to use an effective form of contraception for the duration of the study, or they must have been postmenopausal for at least one year.

Myofascial pain was identified through clinical examination performed by an experienced physician specialized in Physical and Rehabilitative Medicine, based on the presence of active trigger points, taut bands, local tenderness, and referred pain upon palpation.

The exclusion criteria were: (a) significant cognitive impairment (Mini-Mental State Examination score < 24) and/or inability to provide informed consent; (b) ongoing treatment with anti-inflammatory drugs or other rehabilitative therapies; (c) presence of radicular syndromes due to idiopathic, toxic, metabolic, infectious, demyelinating, or neoplastic origin; (d) patients affected by spondylolysis or spondylolisthesis; (e) recent cervical trauma and/or surgery; (f) oncological diseases, rheumatoid arthritis, fibromyalgia, hypothyroidism, or diabetes mellitus; and (g) active infectious diseases. Patients signed their informed consent for data processing after reading the written information sheet and understanding both the contents and the additional oral explanations provided by the study personnel. Participants were informed of their rights under Article 7 of Legislative Decree 30/06/2003, N. 196 (Privacy codice).

Participants were randomly assigned to one of the two treatment groups using a computer-generated simple randomization sequence with a 1:1 allocation ratio. The randomization sequence was generated by an independent researcher who was not involved in participant recruitment, assessment, treatment delivery, or data analysis. Allocation concealment was ensured through sequentially numbered, sealed opaque envelopes prepared by the same independent researcher. Eligible participants were enrolled by the study investigators, and group assignment was performed after baseline assessment by opening the corresponding sealed envelope. In the first phase, all of them underwent 8 sessions of O_2_O_3_ therapy (1/week for 8 weeks). Then, the experimental group underwent a rehabilitation treatment based on the McKenzie method for a total of 10 sessions, while the Control group underwent 10 sessions of rehabilitation treatment based on the Back School. This study was approved by the Ethics Committee of Calabria Region, providing the following code: 78/2024. The protocol was registered on ClinicalTrials.gov with Identifier: NCT07504432. Due to the pilot nature of the study and the expected possibility of dropouts during follow-up, a slightly larger sample than the minimum calculated sample size was recruited. Allocation concealment was ensured through sealed opaque envelopes prepared by an independent researcher. Due to the nature of the rehabilitation interventions, participant and therapist blinding was not feasible. No serious adverse events related to O_2_O_3_ injections or rehabilitation treatments were observed during the study period.

### 2.2. Oxygen–Ozone Therapy

A physician specialized in Physical and Rehabilitative Medicine, with more than 10 years of experience in O_2_O_3_ injections, performed the intramuscular paravertebral O_2_O_3_ therapy.

**Figure 1:**
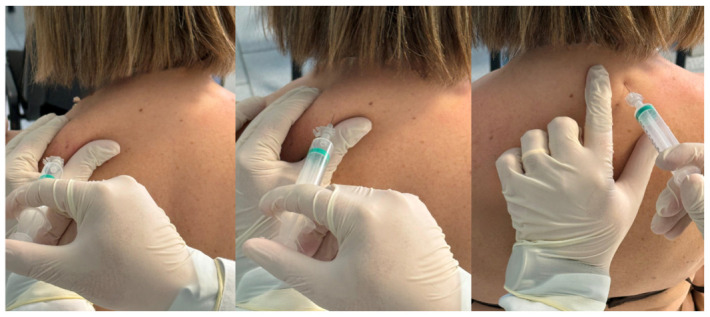
Oxygen–ozone therapy session.

All the participants underwent 8 intramuscular paravertebral cervical injections of O_2_O_3_ gas mixture, using an ozone concentration of 10 μg/mL in a 10 mL syringe, with a 27-gauge × 1/2 needle (0.4 mm × 13 mm). The O_2_O_3_ was obtained through an Ozonline E80 generator (Eco3 s.n.c., Brandizzo, TO, Italy) connected to a pure O_2_ source. The O_3_ generator uses O_2_ through high-voltage tubes and has an O_2_O_3_ output of 5%, ranging from 4000 to 14,000 L. During each treatment session, 5 symmetrical 2 mL injections were administered into the paravertebral cervical muscles. Sessions were repeated once a week for eight weeks (for further details, see Figure 1).

### 2.3. Physical Therapy

#### 2.3.1. McKenzie Group

**Figure 2:**
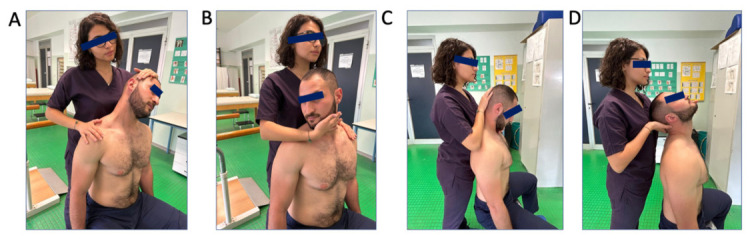
The progressive sequence application of McKenzie exercises. (**A**) Lateral cervical flexion in a sitting position. (**B**) Cervical rotation in a seated position. (**C**) Cervical flexion in a sitting position. (**D**) Cervical extension in a seated position.

The McKenzie method requires active patient participation, encouraging self-treatment in repeated movements and postural positions, which consequently directs the therapeutic plan [25,26,27,28]. The treatment was performed in a sitting position, with the therapist explaining the method and guiding the patient through each exercise. When the patients had difficulty in maintaining the seated position due to increased pain, the treatment was implemented in the supine position, with the head better supported to reduce joint compression and strain. When the pain decreased, the patient came back to the seated position. At the beginning of each session, exercises to improve posture and head retraction were proposed. In the first sessions, the retraction movement was associated with exercises that involved the least restricted and painless movements of the cervical spine in the three planes of motion. Once the centralization and decrease in pain were achieved, exercises were also performed in limited movement areas, as depicted in Figure 2.

#### 2.3.2. Back School Group

The control group underwent cervical rehabilitation following the Back School method. The cervical spine range of motion (ROM) was initially assessed, identifying shortened muscles and painful and restricted movements to plan the personalized exercise plan [29,30]. After properly aligning the patient in a supine position, manual therapy, including superficial and deep muscle kneading, muscle pressure, and friction, was used to promote muscle relaxation and analgesic effects. Subsequently, cervical traction was performed to reduce potential vertebral compression and alleviate pain and enhance muscle relaxation. Passive kinesiotherapy was used to mobilize the head across all three planes of motion: (a) pain-free, unrestricted ROM; (b) pain-free, restricted ROM; (c) painful, restricted ROM and remaining within the pain-free ROM.

**Figure 3:**
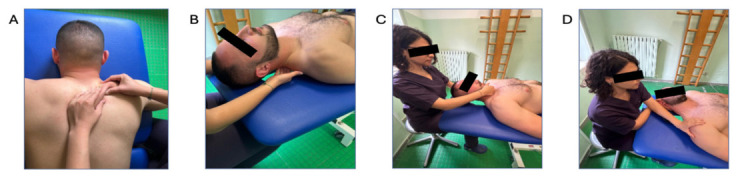
The progressive sequence application of Back school exercises. (**A**) Superficial kneading from the prone position. (**B**) Cervical friction from the supine position (**C**) Cervical traction from supine position. (**D**) Left lateral tilt from supine position.

Finally, the stretching exercises were structured as follows: exercises were performed in the side-lying position to target the lateral compartment of the neck, while self-stretching techniques from a seated or standing position were conducted to target the anterolateral and posterior compartments of the neck (Figure 3).

### 2.4. Outcome Measures

The primary outcome was the evaluation of disability related to neck pain, using the Neck Disability Index (NDI) questionnaire, which is currently the most widely used tool for the neck pain disability evaluation [31]. The NDI questionnaire is a self-administered questionnaire composed of 10 sections, addressing pain intensity and its impact on activities of daily living (ADL). Each section offers six possible answers, scored from 0 (no difficulty or pain) to 5 (unable to perform the activity or severe pain). The score is calculated using the following formula: (sum of the scores of all completed sections)/(total possible score) × 100. If all sections are completed, the maximum possible score is 50 points. If one section is left incomplete, the total possible score is reduced to 45 points [32].

Secondary outcomes were: (a) the Numerical Rating Scale (NRS); (b) EuroQol-5D; and (c) EQ-VAS. NRS assesses pain symptoms through a unidimensional 11-point scale that measures pain intensity in adults. The scale consists of a horizontal line ranging from 0 (“no pain”) to 10 (“worst imaginable pain”). The health-related quality of life was measured using the EuroQol questionnaire. It is divided into two parts. The first is EuroQol-5D, which includes the following five dimensions: mobility, self-care, usual activities, pain/discomfort, and anxiety/depression. Each dimension has three levels: no problems, some problems, and extreme problems. Patients indicate their health status by ticking the box next to the statement that best describes their condition in each of the five dimensions. The second is EQ-VAS, which records the patient’s self-rated health on a vertical VAS, with endpoints labeled “best imaginable health state” and “worst imaginable health state.”

Patients were evaluated before the start of O_2_O_3_ injections (T0); after O_2_O_3_ injections, at 8 weeks (T1); at the end of physical therapy treatments (T2); and during a follow-up evaluation at 24 weeks from baseline (T3).

### 2.5. Statistical Analysis

Continuous variables and parametric data were expressed as means and standard deviation. Repeated measures ANOVA was used to analyze differences along timepoints. Moreover, at each timepoint, outcome measure differences were assessed within each group at T0, T1, T2, and T3 with paired *t*-test, and between groups with independent *t*-test. A *p*-value < 0.05 was considered statistically significant. Statistical analysis was performed using Jamovi, version 2.4. The G-Power statistics module from Jamovi software (2.4.14) was used to estimate the proper sample size on the primary outcome (NDI). We considered an alpha level of 0.05 with 80% power and a minimum effect size of 0.40. Thus, through a repeated-measures analysis of variance of group relations, a fit sample size was established to be 20 subjects per group. The repeated-measures ANOVA model included time as the within-subject factor and treatment group as the between-subject factor.

## 3. Results

**Figure 4:**
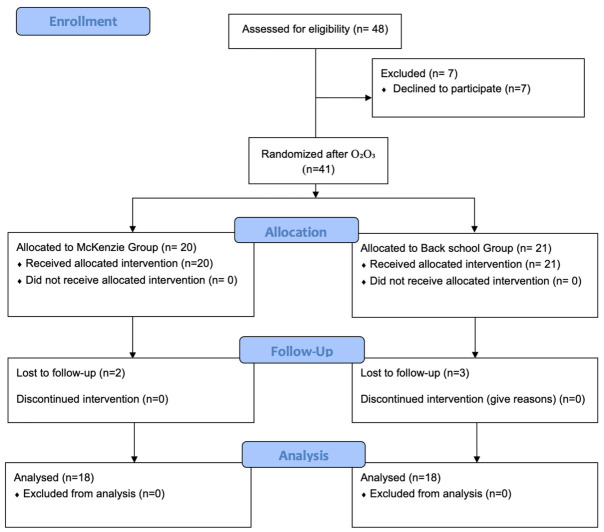
Consort flow diagram.

In this prospective double-arm pilot study, 48 patients were assessed for eligibility. After the exclusion of 7 participants who declined to participate, 41 patients were randomized in a 1:1 ratio into two groups: the McKenzie group (n = 20) and the Back School group (n = 21). During follow-up, two participants in the McKenzie group and three participants in the Back School group were lost to follow-up due to personal and logistical reasons and poor adherence to the therapeutic program. Therefore, 36 patients completed the study and were included in the final analysis (McKenzie group, n = 18; Back School group, n = 18). The Back School group consisted of 21 patients at baseline: 8 males (38.10%) and 13 females (61.90%), with a mean age of 63.9 ± 13.42 years and a BMI of 24.02 ± 2.77 kg/m^2^. The McKenzie group consisted of 20 patients: 3 males (15%) and 17 females (85%), with a mean age of 57.3 ± 12.88 years and a BMI of 24.8 ± 2.72 kg/m^2^ (Figure 4). The demographic characteristics of the study participants are reported in Table 1.

At T0, there were no intergroup differences for all the outcomes analyzed. The demographic characteristics of the study participants are reported in Table 1.

At T1, we found a statistically significant improvement compared to T0 for all the outcomes analyzed. For the NDI and NRS, both groups showed a lower score. NDI: Control group (T0: 42.6 ± 18.2 vs. T1: 27.9 ± 14.2; *p* < 0.001) and the McKenzie group (T0: 44.3 ± 15.99 vs. T1: 27.6 ± 12.13; *p* < 0.001); NRS: Control group (T0: 8.04 ± 1.80 vs. T1: 5.42 ± 2.15; *p* < 0.001) and McKenzie group (T0: 8.4 ± 1.66 vs. T1: 5.50 ± 1.9; *p* < 0.001). We found a higher score for the EQ-5D-3L: Control group (T0: 0.498 ± 0.198 vs. T1: 0.709 ± 0.175; *p* < 0.001) and McKenzie group (T0: 0.494 ± 0.222 vs. T1: 0.708 ± 0.31; *p* < 0.001); and EQ-VAS: McKenzie group (T0: 0.530 ± 0.208 vs. T1: 0.688 ± 0.163; *p* < 0.001) compared to the Control group (T0: 0.483 ± 0.195 vs. T1: 0.598 ± 0.165; *p* = 0.027).

At T2, after the end of rehabilitation interventions, the McKenzie and Control groups maintained a statistically significant improvement in all the outcomes analyzed: NDI (McKenzie group T2 14.5 ± 8.6; *p* < 0.001 vs. Control group T2 14.5 ± 12.30; *p* < 0.001); NRS (McKenzie group T2 3.15 ± 1.9; *p* < 0.001 vs. Control group T2 2.04 ± 1.7; *p* < 0.001); EQ-VAS (McKenzie group T2 0.800 ± 0.163; *p* < 0.001 vs. Control group T2 0.711 ± 0.195; *p* < 0.001); EQ5D3L (McKenzie group T2 0.799 ± 0.166; *p* = 0.010 vs. Control group T2 0.715 ± 0.183; *p* = 0.009). The improvements achieved at T2 were maintained in all outcomes measured at T3 (see Table 2 for further details).

Our results were further confirmed by the repeated measures ANOVA test, where we found no statistically significant values.

There were no side effects in the entire sample at all the timepoints.

## 4. Discussion

This study aimed to evaluate the effects of a combined treatment of O_2_O_3_ injections and physiotherapy exercises, based on the McKenzie and Back School method, for disability reduction and pain relief in patients with chronic non-specific neck pain.

The primary outcome explored was the NDI, which assesses pain intensity and its impact on ADL. At the end of the O_2_O_3_ injection cycle, both groups showed a statistically significant improvement in pain relief, as also highlighted by the significant improvements observed in the NDI scale. Indeed, O_2_O_3_ injections provide anti-inflammatory and antioxidant effects through cytokine and prostaglandin modulation, as well as the enhancement of superoxide dismutase, which might manage various musculoskeletal disorders, including spinal issues such as neck pain and LBP, as reported in a recent comprehensive review [11,12,13,14,15,16,17,18,19,20].

It should be noted that the observed treatment effects were largely attributable to the O_2_O_3_ therapy phase. Following O_2_O_3_ treatment, both groups showed significant reductions in pain intensity, as measured by the NRS (Back School: 8.04 ± 1.80 vs. 5.42 ± 2.15, *p* < 0.001; McKenzie: 8.40 ± 1.66 vs. 5.50 ± 1.90, *p* < 0.001), together with significant improvements in disability, as assessed by the NDI (McKenzie: 44.3 ± 15.99 vs. 27.6 ± 12.13, *p* < 0.001; Back School: 42.6 ± 18.2 vs. 27.9 ± 14.2, *p* < 0.001). Indeed, all participants underwent the same O_2_O_3_ treatment in the first part of the treatment, representing a potential limitation of the study design. Nevertheless, after the physiotherapy (independently from the approach), there was another significant improvement, without significant between-group differences.

In this scenario, pain relief induced by O_2_O_3_ therapy and physiotherapy could facilitate the execution of ADLs, as evidenced by the improvements observed in the secondary outcomes, testifying the need of a combination of injections and physiotherapy in these complex patients.

Our findings are consistent with the recent review by Jandura et al. [33], which proved significant improvements in pain and decreases in disability, as assessed by NDI and NRS. Furthermore, our findings are supported by the retrospective observational study of Ucar et al. [34], which demonstrated the role of intramuscular oxygen–ozone therapy in the pain management of 72 patients specifically affected by neck pain. Their findings, based on cervical paravertebral injections, provided further evidence for the potential analgesic effects of O_2_O_3_ in this patient population. In 2022, a prospective study involving 540 patients with cervicobrachial pain obtained a significant reduction in the NRS score after 11 weekly cervical intramuscular O_2_O_3_ injections, maintaining pain relief at long-term follow-up [35]. Similar results were obtained in a retrospective study of 156 patients affected by cervicobrachial pain, showing significant improvement in pain and disability, as measured at many timepoints [36].

Nonetheless, further functional improvements were observed in both the McKenzie and BackSchool groups after completing the cycle of physical therapies. Both groups showed a significant improvement in the NDI and NRS at the end of the treatment and at the 24-week follow-up. However, both the McKenzie and Back School approaches resulted in significant improvements in pain and disability, with no significant between-group differences detected [37].

These findings may be explained by the specific approach of the McKenzie method, which required active participation of the patients during the exercises via self-treatment strategies, engaging in self-directed repeated movements and sustained positions aimed at reducing pain and disability and improving spine mobility [21]. This type of approach seemed to allow patients to gain greater awareness of their functional disorders, helping them to internalize appropriate strategies that can also be applied during ADL activities, thereby reducing disability [22]. A recent RCT of Abdel-Aziem et al. [27] showed that McKenzie exercises were more effective than conventional physiotherapy, concerning deep neck flexor strength in treating neck pain, functional disability, and mobility. Another experimental study on 120 patients highlighted that the McKenzie protocol is more useful than segmental spinal stabilization exercises in treating neck pain in people with cervical postural disorder [25]. A systematic RCTs review by Baumann et al. analyzed how McKenzie exercises could provide clinically and statistically significant pain improvement in moderate-to-severe neck pain, with slight superiority over other physiotherapy approaches [26].

Regarding secondary outcomes, the EuroQol 5D-3L was used to evaluate quality of life in neck pain related-disability. Both the McKenzie and Control groups showed statistically significant improvements after O_2_O_3_ injections and associated physical therapies. These positive findings persisted at the 24-week follow-up, with persistent improvements in both groups. Indeed, the improvement in cervical functionality and the reduction in pain have been shown to have a positive impact on the quality of life of people with chronic non-specific neck pain (with significant improvements observed in terms of EQ-VAS).

Therefore, in the post-COVID-19 a combined therapeutic approach involving O_2_O_3_ injections and physiotherapy might reduce disability and provide pain relief for patients with chronic non-specific neck pain, providing evidence for how the rehabilitation is crucial in subjects with specific rehabilitative needs [38,39,40,41,42,43].

However, it is important to recognize the presence of several study limitations. First, there is a lack of objective assessments due to evaluations relying on self-reported outcome measures and therefore being based on the patient’s subjective perception. Second, in the first phase of the study, all the patients underwent the same O_2_O_3_ treatment, and the differences between groups indicate no clinical significance. Furthermore, there is an absence on baseline characteristics such as pain duration, occupational status, prior treatments, and comorbidities, which would have strengthened the clinical interpretation of these findings, providing higher external validity. Moreover, the absence of blinding should also be considered a limitation of this study with respect to the strength of our protocol. Lastly, the absence of isolated O_2_O_3_-only and physical therapy-only groups prevents definitive conclusions being reached regarding the superiority of combined interventions.

## 5. Conclusions

Taken together, this prospective double-arm pilot study demonstrated the effectiveness of a combined treatment of cervical intramuscular paravertebral O_2_O_3_ injections and two different physiotherapy approaches, namely, McKenzie and Back School, in disability and pain management in people affected by chronic non-specific neck pain.

In particular, both the McKenzie and Back School approaches were associated with improvements in pain relief, cervical function, disability, and quality of life. No statistically significant differences were observed between treatment groups.

To the best of our knowledge, this is the first study in the literature combining different physiotherapy treatments after sessions of O_2_O_3_ therapy injections to treat neck pain. Thus, future RCTs with larger sample sizes and longer follow-ups are necessary to confirm these findings.

## Figures and Tables

**Table 1:** Population characteristics.

	Control Group	McKenzie
Male/Female	n = 8 (38.10%); n = 13 (61.90%)	n = 3 (15%); n = 17 (85%)
Age (years)	63.9 ± 13.42	57.3 ± 12.88
BMI (kg/m^2^)	24.02 ± 2.77	24.8 ± 2.72

**Table 2:** Independent samples *t*-test (Mann–Whitney U) and repeated measures ANOVA test for the outcomes of the McKenzie (MCK) and Control (CP) groups. The repeated-measures ANOVA model included time as the within-subject factor and treatment group as the between-subject factor.

		T0	T1	∆T0–T1	T2	∆T1–T2	∆T0–T2	T3	∆T2–T3	∆T0–T3	Repeated Measures ANOVA
				*p* Value		*p* Value	*p* Value		*p* Value	*p* Value	
NRS	Back School	8.04 ± 1.80	5.42 ± 2.15	*p* < 0.001	2.04 ± 1.7	*p* < 0.001	*p* < 0.001	2.58 ± 1.73	*p* = 0.473	*p* ≤ 0.001	*p* = 0.408
	McKenzie	8.4 ± 1.66	5.50 ± 1.9	*p* < 0.001	3.15 ± 1.9	*p* < 0.001	*p* < 0.001	3.18 ± 2.27	*p* = 0.849	*p* ≤ 0.001	
	Between group *p*-value	*p* = 0.520	*p* = 0.850		*p* = 0.220			*p* = 0.394			
NDI	Back School	42.6 ± 18.2	27.9 ± 14.2	*p* < 0.001	14.05 ± 12.30	*p* ≤ 0.001	*p* ≤ 0.001	14.9 ± 10.4	*p* = 0.961	*p* ≤ 0.001	*p* = 0.701
	McKenzie	44.3 ± 15.99	27.6 ± 12.13	*p* < 0.001	14.5 ± 8.6	*p* ≤ 0.001	*p* ≤ 0.001	18.6 ± 12.05	*p* = 0.160	*p* ≤ 0.001	
	Between group *p*-value	*p* = 0.751	*p* = 0.946	*p* < 0.001	*p* = 0.985			*p* = 0.364			
EQ5D3L	Back School	0.498 ± 0.198	0.709 ± 0.175	*p* < 0.001	0.715 ± 0.183	*p* = 0.009	*p* < 0.001	0.804 ± 0.161	*p* = 0.848	*p* < 0.001	*p* = 0.822
	McKenzie	0.494 ± 0.222	0.708 ± 0.31	*p* = 0.001	0.799 ± 0.166	*p* = 0.010	*p* < 0.001	0.773 ± 0.09	*p* = 0.266	*p* < 0.001	
	Between group *p*-value	*p* = 0.948	*p* = 0.973		*p* = 0.910			*p* = 0.511			
EQVAS	Back School	0.483 ± 0.195	0.598 ± 0.165	*p* = 0.027	0.711 ± 0.195	*p* < 0.001	*p* < 0.001	0.721 ± 0.186	*p* = 0.884	*p* ≤ 0.001	*p* = 0.283
	McKenzie	0.530 ± 0.208	0.688 ± 0.163	*p* < 0.001	0.800 ± 0.125	*p* = 0.004	*p* < 0.001	0.753 ± 0.143	*p* = 0.110	*p* < 0.001	
	Between group *p*-value	*p* = 0.463	*p* = 0.088		*p* = 0.095			*p* = 0.580			

## Data Availability

Data can be obtained from the corresponding author upon reasonable request.

## Abbreviations

Activities of Daily Living (ADL); Body Mass Index (BMI); EuroQol (EQ5D3L); EuroQol–Visual Analogical Scale (EQ-VAS); Neck Disability Index (NDI); Non-Steroidal Anti-Inflammatory Drugs (NSAIDS); Numerical Rating Scale (NRS); Oxygen–Ozone (O_2_O_3_); Range Of Motion (ROM); Randomized Controlled Trial (RCT).
